# Portrait of Matrix Gene Expression in *Candida glabrata* Biofilms with Stress Induced by Different Drugs

**DOI:** 10.3390/genes9040205

**Published:** 2018-04-10

**Authors:** Célia F. Rodrigues, Mariana Henriques

**Affiliations:** Laboratório de Investigação em Biofilmes Rosário Oliveira (LIBRO), Centre of Biological Engineering, University of Minho, 4710-057 Braga, Portugal; rodriguescf@ceb.uminho.pt

**Keywords:** *Candida*, biofilms, matrix, drug resistance, gene expression, *Candida glabrata*

## Abstract

(1) Background: *Candida glabrata* is one of the most significant *Candida* species associated with severe cases of candidiasis. Biofilm formation is an important feature, closely associated with antifungal resistance, involving alterations of gene expression or mutations, which can result in the failure of antifungal treatments. Hence, the main goal of this work was to evaluate the role of a set of genes, associated with matrix production, in the resistance of *C. glabrata* biofilms to antifungal drugs. (2) Methods: the determination of the expression of *BGL2*, *XOG1*, *FKS1*, *FKS2*, *GAS2*, *KNH1*, *UGP1*, and *MNN2* genes in 48-h biofilm’s cells of three *C. glabrata* strains was performed through quantitative real-time PCR (RT-qPCR), after contact with Fluconazole (Flu), Amphotericin B (AmB), Caspofungin (Csf), or Micafungin (Mcf). (3) Results: Mcf induced a general overexpression of the selected genes. It was verified that the genes related to the production of β-1,3-glucans (*BGL2*, *XOG1*, *GAS2*) had the highest expressions. (4) Conclusion: though β-1,6-glucans and mannans are an essential part of the cell and biofilm matrix, *C. glabrata* biofilm cells seem to contribute more to the replacement of β-1,3-glucans. Thus, these biopolymers seem to have a greater impact on the biofilm matrix composition and, consequently, a role in the biofilm resistance to antifungal drugs.

## 1. Introduction

Fungal infections continue to increase worldwide, particularly among immunosuppressed patients, individuals under prolonged hospitalization, catheterization, or continued antimicrobial treatments [1,2,3]. *Candida* spp. are the commonest fungal species involved in these diseases. *Candida albicans* is the most isolated species, but *Candida glabrata* and *Candida parapsilosis* are the second most isolated species in the United States of America and Europe, respectively [1,4,5]. Though *C. glabrata* does not have the capacity to form hyphae and pseudohyphae or to secret proteases, this species has other virulence factors, such as the ability to secrete phospholipases, lipases, and haemolysins and, importantly, the capacity to form biofilms [6,7,8]. These factors highly contribute to a high aggressiveness, resulting in a low therapeutic response and severe cases of recurrent candidiasis [8,9]. Biofilms are communities of microorganisms that colonize tissues and indwelling medical devices, embedded in an extracellular matrix [10,11]. These heterogeneous structures provide high resistance to antifungal therapy and strong host immune responses [7,8,12]. *C. glabrata* has shown to form a compact biofilm structure in different multilayers [6,7], with proteins, carbohydrates, and ergosterol into their matrices [6,7,13].

Various reports have shown the presence of β-1,3 glucans in the biofilm matrices of *C. albicans* [14,15,16,17]. Interestingly, it has been demonstrated that an increase in cell wall glucan was associated with biofilm growth [14] and, more recently, β-1,3 glucans were shown to be also present in the matrices of *C. glabrata* biofilms [13,18,19]. This specific carbohydrate has been associated with a general increase of extracellular matrix delivery, which is critical for securing biofilm cells to a surface and crucial to develop an antifungal drug resistance phenotype [14,19,20,21,22,23]. Several genes are involved in the delivery and accumulation of extracellular matrix. It is recognized that, in *C. albicans*, the major β-1,3 glucan synthases are encoded mainly by *FKS1* but also by *FKS2* [24]. The *BGL2* and *XOG1* genes also have important roles in glucan matrix delivery by encoding glucanosyltransferases and β-1,3 exoglucanase, respectively [25,26]. These genes play an important part in cell wall remodeling, however, the influence of the corresponding enzymes in matrix glucan delivery does not appear to affect cell wall ultrastructure or β-1,3 glucan concentration, suggesting that these enzymes function specifically for matrix delivery [17,19,26,27,28]. Identical to *Saccharomyces cerevisiae*, in *C. glabrata*, the *GAS* gene family is a regulator of the production of β-1,3 glucan [29]. *Gas2*, a glycosylphosphatidylinositol (GPI)-anchored cell surface protein [30,31], is a putative carbohydrate-active enzyme that may change cell wall polysaccharides [29,32].

Another carbohydrate of *C. glabrata* cell wall is β-1,6-glucan, present as a polymer covalently attached to glycoproteins [33,34,35,36], β-1,3-glucan, and chitin [37]. Nagahashi et al. [36] reported the isolation of *KNH1* homologs (genes encoding cell surface *O*-glycoproteins), suggesting the evolutionary conservation of these molecules as essential components of β-1,6-glucan synthesis in *C. glabrata,* which was also discussed before [35,38]. Additionally, the *UGP1* gene is a putative uridine diphosphate (UDP)-glucose pyrophosphorylase related to the general β-1,6-d-glucan biosynthetic process [39,40]. During stress conditions, several *S. cerevisiae* orthologous genes are induced in *C. glabrata*. In glucose starvation stress, *UGP1* is induced [39].

The external layer cell wall of *Candida* spp. also consists of highly glycosylated mannoproteins [41,42,43], which play a major role in host recognition, adhesion, and cell wall integrity [44,45,46,47,48,49,50,51,52,53,54,55,56]. These proteins have both *N*- and *O*-linked sugars, predominantly mannans, which are also known to be present in the biofilm matrices of *C. albicans* [57,58,59]. The *MNN2* gene is one putative element of *N*-linked glycosylation, directly responsible for mannans production for both cell and biofilm matrices of *C. glabrata* [57,58,59].

The goal of this work was to determine the expression profile of selected genes (Table 1 and Table 2) related to the production of biofilm matrix components in response to stress caused by drugs from the most important antifungal classes: azoles (Fluconazole, Flu), polyenes (Amphotericin B, AmB), and echinocandins (Caspofungin, Csf, and Micafungin, Mcf).

## 2. Materials and Methods

### 2.1. Organisms

Three strains of *C. glabrata* were used in the course of this study: One reference strain (*C. glabrata* ATCC 2001) from the American Type Culture Collection (Manassas, VA, USA), one strain recovered from the urinary tract (*C. glabrata* 562123) of a patient, and one strain recovered from the vaginal tract of a patient (*C. glabrata* 534784) in the Hospital Escala, Braga, Portugal. The identity of all isolates was confirmed using CHROMagar^TM^ *Candida* (CHROMagar^TM^, Paris, France) and by PCR-based sequencing using specific primers (*ITS1* and *ITS4*) against the 5.8 s subunit gene reference [60]. The PCR products were sequenced using the ABI-PRISM Big Dye terminator cycle sequencing kit (Perkin Elmer, Applied Biosystems, Warrington, UK).

### 2.2. Growth Conditions

For each experiment, *C. glabrata* ATCC2001, *C. glabrata* 534784, and *C. glabrata* 562123 strains were subcultured on Sabouraud dextrose agar (SDA) (Merck, Darmstadt, Germany) for 24 h at 37 °C. The cells were then inoculated in Sabouraud dextrose broth (SDB) (Merck) and incubated for 18 h at 37 °C under agitation at 120 rpm. After incubation, the cells were harvested by centrifugation at 3000× *g* (Thermo Scientific, CL10, Hampton, NH, USA) for 10 min at 4 °C and washed twice with phosphate buffered saline (PBS, pH = 7.5). The cell pellets were then suspended in Roswell Park memorial institute (RPMI), and the cellular density was adjusted to 1 × 10^5^ cells/mL, using a Neubauer counting chamber.

### 2.3. Antifungal Drugs

Flu, Csf, and Mcf were kindly provided by Pfizer^®^ (New York, NY, USA), MSD^®^ (Kenilworth, NJ, USA) and Astellas Pharma, Ltd., (Tokyo, Japan), respectively, in their pure form. AmB was purchased from Sigma^®^ (Sigma-Aldrich, Buffalo, NY, USA). Aliquots of 5000 mg/L were prepared using dimethyl sulfoxide (DMSO). The final concentrations used were prepared in RPMI-1640 (Sigma-Aldrich).

### 2.4. Biofilm Formation

The minimum biofilm eradicatory concentration (MBEC) values were previously determined by the group, according to the European committee on antimicrobial susceptibility testing (EUCAST) guidelines [61,62]. For biofilm formation, standardized cell suspensions (1000 μL) were placed into selected wells of 24-wells polystyrene microtiter plates (Orange Scientific, Braine-l’Alleud, Belgium). At 24 h, 500 μL of RPMI-1640 was removed, and an equal volume of fresh RPMI-1640 plus the antifungal solution was added, on the basis of the MBEC values determined and indicated in bold in Table 1 (2× concentrated). The plates were incubated at 37 °C for additional 24 h at 120 rpm. RPMI-1640 containing only the antifungal agent was used as a negative control. As a positive control, cell suspensions were tested in the absence of the antifungal agent [18].

### 2.5. Gene Expression Analysis

#### 2.5.1. Gene Selection and Primer Design for Quantitative Real-Time PCR

Genes related to the production of biofilm matrix components (β-1,3, β-1,6 glucans, and mannans)—*BGL2*, *FKS1*, *FKS2*, *GAS2*, *KNH1*, *UGP1*, *XOG1 and MNN2*—were selected for this study. The gene sequences of interest were obtained from *Candida* Genome Database [63] and the primers for quantitative real-time PCR (RT-qPCR) were designed using Primer 3 [64] web-based software and are listed in Table 2. *ACT1* was chosen as a housekeeping gene. In order to verify the specificity of each primer pair for its corresponding target gene, the PCR products were first amplified from *C. glabrata* ATCC2001.

#### 2.5.2. Preparation of Biofilm Cells for RNA Extraction

After biofilm formation, the medium was eliminated, and the wells were washed with sterile water to remove non-adherent cells. The biofilms were scraped from the wells with 1 mL of sterile water and sonicated (Ultrasonic Processor, Cole-Parmer, IL, USA) for 30 s at 30 W to separate the cells from the biofilm matrix. The cells were harvested by centrifugation at 8000× *g* for 5 min at 4 °C [18].

#### 2.5.3. RNA Extraction

RNA extraction was performed using PureLink RNA Mini Kit (Invitrogen, Carlsbad, CA, USA). Prior to RNA extraction, a lysis buffer from PureLink RNA Mini kit was prepared by adding 1% of *ß*-mercaptoethanol to the supplied buffer solution. Then, 500 µL of lysis buffer containing glass beads (0.5 mm diameter) was added to each pellet. The cell suspensions were homogenized twice for 30 s using a Mini-Bead-Beater-8 (Stratech Scientific, Soham, UK). After cell disruption, the PureLink RNA Mini Kit (Invitrogen) was used for total RNA extraction according to the manufacturer’s recommended protocol. To avoid potential DNA contamination, the samples were treated with RNase-Free DNase I (Invitrogen) [18].

#### 2.5.4. Synthesis of Complementary DNA

To synthesize complementary DNA (cDNA), the iScript cDNA Synthesis Kit (Bio-Rad, Hercules, CA, USA) was used according to the manufacturer’s instructions. For each sample, 10 μL of the extracted RNA was used in a final reaction volume of 50 μL. cDNA synthesis was performed firstly at 70 °C for 5 min and then at 42 °C for 1 h. The reaction was stopped by heating for 5 min at 95 °C [18].

#### 2.5.5. Quantitative Real-Time PCR

RT-qPCR (CFX96 Real-Time PCR System, Bio-Rad) was performed to determine the relative levels of all genes mRNA transcripts in the RNA samples, with *ACT1* used as a reference *Candida* housekeeping gene. Each reaction mixture consisted of a working concentration of SoFast EvaGreen Supermix (Bio-Rad), 50 µM forward and reverse primers, and 4 µL cDNA, in a final reaction volume of 20 µL. Negative controls (water) as well as non-transcriptase reverse controls (NRT) were included in each run [18]. The relative quantification of gene expression was performed by the 2^−ΔC_T_^ method [65]. Each reaction was performed in triplicate, and mean values of relative expression were determined for each gene. The results are presented after calculation of 2^−ΔC_T_^.

### 2.6. Statistical Analysis

All experiments were repeated three times in independent assays. The results were compared using one-way analysis of variance (ANOVA), Dunnett’s multiple comparisons tests, using GraphPad^TM^ Prism^TM^ 7 software (GraphPad Software, San Diego, CA, USA). All tests were performed with a confidence level of 95%. In order to determine the similarity of the strains’ gene profiles, the Pearson Correlation Coefficient (r) was also applied.

## 3. Results and Discussion

Candidaemia related to *C. glabrata* has been increasing in the last years in parallel with its high drug resistance, particularly to the azole antifungal class [1,20,66]. Biofilms of *C. glabrata* are highly recalcitrant to treatments with antifungal agents as a consequence of multiple resistance mechanisms, such as those linked to the presence of a strong net of exopolysaccharydes and other biopolymers that protect the cells and hinder the diffusion of the drugs [1,15,67,68,69]. In order to stress *C. glabrata* biofilm cells, four antifungals were applied (at concentrations based on MBECs values, Table 1) in pre-formed biofilms, and then an evaluation of biofilms’ matrix gene expression was performed and compared with the expression of a housekeeping gene.

**Figure 1:**
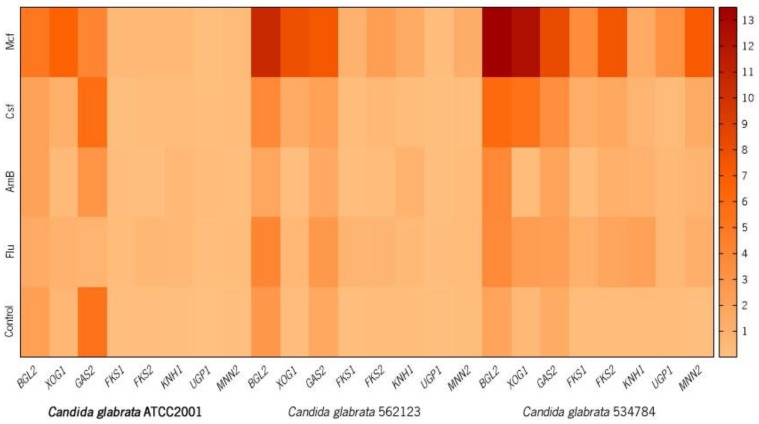
Real-time PCR expression profiling of *BGL2*, *XOG1*, *GAS2*, *FKS1*, *FKS2*, *KNH1*, *UGP1*, and *MNN2* genes in biofilm cells of *Candida glabrata* ATCC2001 (reference strain, in bold) (a), *C. glabrata* 562123 (b), and *C. glabrata* 534784 (c) in the presence of antifungal drugs. The heatmap was generated by a log transformation of the RT-qPCR data and the fold-change (FC) expression determined through 2^−ΔC_T_^. The numerical scale on the right represents the FC. (Control: non-treated cells).

Figure 1 shows the heatmap with the results of the RT-qPCR expression profiling of biofilm cells of *C. glabrata* ATCC2001 (A), *C. glabrata* 562123 (B), and *C. glabrata* 534784 (C) in the presence of antifungal drugs. The final data are presented in fold-change (FC) in comparison to the expression of the housekeeping gene (2^−ΔC_T_^) [70].

Generally, *BGL2*, *FKS1*, *FKS2*, *GAS2*, and *XOG1* displayed higher expression levels in biofilm cells in response to the drugs and, by contrast, *KNH1*, *UPG1*, and *MNN2* displayed minor expression changes (Figure 1 and Table 3).

*BGL2* showed similar expression in the control groups, and its FC expression decreased in the reference strain when Flu and Csf were present (FC: 1.40 and 2.00 respectively) and in the urinary strain when AmB was present (FC: 1.71). In all the other cases, *BGL2* FC expression increased, particularly when the biofilms were treated with Mcf (FC: *C. glabrata* ATCC2001: 5.13; *C. glabrata* 562123: 10.58; *C. glabrata* 534784: 13.49). All changes in *BGL2* expression were statistically significant compared to the untreated cells (*p* < 0.0001). Compared to the controls, *XOG1* gene revealed a statistically significant downregulation after contact with AmB (FC: *C. glabrata* ATCC2001 0.37; *C. glabrata* 562123 0.10; *C. glabrata* 534784: 0.27) and overexpression in the presence of all the other antifungals, in all strains. For this gene, the most noteworthy overexpression was observed in Flu-treated *C. glabrata* 534784 (FC: 2.35, *p* < 0.0001), Csf-treated *C. glabrata* 562123 and *C. glabrata* 534784 (FC: 1.42 and 5.45, *p* < 0.0001, respectively), and in all strains after contact with Mcf (FC: *C. glabrata* ATCC2001: 6.54, *C. glabrata* 562123: 7.89, *C. glabrata* 534784: 12.38, all *p* < 0.0001).

In an important report, Taff et al. [19] concluded that mutants of *C. albicans* unable to produce Bgl2 and Xog1 enzymes did not show perturbations in the cell wall glucan composition of biofilm cells, and that these enzymes were not necessary for filamentation or biofilm formation. However, the biofilms had a reduced matrix glucan content, reduced total matrix biomass accumulation, and improved susceptibility to antifungal drug therapy [19]. Similarly, Li et al. [71] showed that, in *C. albicans’* persister cells (frequent in biofilms [72,73]), there was an increased expression of cell wall integrity proteins such as Xog1 and Bgl2. These studies recognized a biofilm-specific pathway involving Bgl2 and Xog1 (and Phr1) enzymes and affecting matrix delivery, by which these enzymes release and modify cell wall glucan for deposition in the extracellular space; however, an alternative explanation is that these enzymes act in the extracellular space, being crucial for mature matrix organization and function [19]. These enzymes have been localized in the cell wall, supporting the hypothesis of their activity in the cell wall, but have also secretion sequences that support an extracellular function. As seen earlier, *BGL2* is one of the glucan modifying genes for glucan delivery, and *XOG1* is a glucanase [19], necessary for modification and delivery of carbohydrates to the mature biofilm matrix. Without delivery and accumulation of matrix glucan, the biofilms exhibit enhanced susceptibility to antifungal drugs [19]. The change in the regulation of *BGL2* and *XOG1* in the biofilm cells of *C. glabrata* after drug treatment that we observed is interpreted as a response of the biofilm cells to the reduction of biofilm matrix, specifically of β-1,3-glucans, and it has been described before [7,13,18].

The *GAS* gene family is also a regulator in the production of β-1,3-glucan, and Gas2 is a glycosylphosphatidylinositol (GPI)-anchored cell surface protein [31] involved in the production of β-1,3-glucan in *C. glabrata* [29,30]. Gas2 is a documented putative carbohydrate-active enzyme and consequently it can alter the cell wall polysaccharides in order to build and remodel the cell wall glycan network during growth in *C. glabrata* [29]. In *C. glabrata* ATCC2001, *GAS2* was highly expressed in the non-treated group and after Csf contact (FC: 5.72), while Flu, AmB, and Mcf led to its downregulation. The clinical isolates upregulated the gene in all conditions, except for AmB treatment of *C. glabrata* 562123 (Figure 1 and Table 3, all *p* < 0.0001). Hence, when analyzing the results of *C. glabrata* 562123 and *C. glabrata* 534784, the *GAS2* network seems to be activated also after glycan’s loss following drug treatment, in order to replace the lack of 1,3-β-glucans and re-establish biofilm cells’ homeostasis. All results compared with those from untreated cells (controls) were statistically significant (*p* < 0.0001).

The resistance to echinocandins increased from 4.9% to 12.3% between 2001 and 2010 [74] with a rapid development of *FKS* mutations in *Candida* spp., especially in *C. glabrata* [75,76]. The amino acid substitutions occurring in *FKS1* [30,77,78,79] and *FKS2* [30,80] are directly related to the resistance to this class of drugs: acquired *FKS* mutations [81] are reported to confer low β-(1,3)-d-glucan synthase sensitivity and to increase the minimum inhibitory concentration (MIC) values, which are related to clinical failure [82]; intrinsic *FKS* mutations, also lead to elevated MIC levels but have a weaker effect on the reduction of β-(1,3)-d-glucan synthase sensitivity [82,83,84,85]. Generally, in the *C. glabrata* strains, a 24 h contact with both echinocandins upregulated *FKS1* and *FKS2* genes and, in the reference strain, the presence of Mcf upregulated *FKS1*. More specifically, the results showed that all strains upregulated the expression of *FKS1* after drug exposure (statistically significant), with the exception of Csf in *C. glabrata* ATCC2001 (FC: 0.07 *p* < 0.001) and Csf in *C. glabrata* 562123 (FC: 0.08; *p* < 0.0001). For *FKS2*, its overexpression was observed for almost all treatments in the three strains, excluding following AmB treatment in *C. glabrata* ATCC2001 (FC: 0.28; *p* < 0.001) and *C. glabrata* 562123 (FC: 0.55; *p* < 0.05). *C. glabrata* 534784 revealed, again, to have the highest capacity to overexpress both genes in response to drug stress. These differences among the strains may be related to the described *Candida* spp. intra-strains variations [62]. Bizerra et al. [76] reported the occurrence of a mutation associated with the resistance phenotype against echinocandins in *C. glabrata* isolated from a single cancer patient with candidemia exposed to antifungal prophylaxis with Mcf. Arendrup et al. [86] revealed that Mcf MICs of *C. glabrata FKS* hot spot mutant isolates were less raised than those obtained for the other echinocandins, showing that the efficacy of Mcf could be differentially dependent on specific *FKS* genes mutations. These reports mention singularities regarding the *FKS* gene and Mcf, which can also be observed in our results (Figure 1 and Table 3). Interestingly, up and downregulations of *FKS1* and *FKS2* were similar in the clinical isolates and parallel to those observed for *BGL2*, which makes sense, since this gene has shown to perform, with *XOG1* (and *PHR1*), in a complementary manner in order to distribute the matrix downstream of the primary β-1,3 glucan synthase encoded by *FKS1* [19]. Previous investigations also found elevated transcript levels of *FKS1*, *BGL2*, and *XOG1* during in vivo *C. albicans* biofilm growth when compared to planktonic growth, which is consistent with our results and with a role in a biofilm-specific function, such as matrix formation [87,88].

The overexpressed values obtained for *BGL1*, *XOG1*, *FKS1*, *FKS2*, and *GAS2* after the stress conditions induced by most antifungals endorse the impact of β-1,3-glucans in the maintenance of the cell and biofilm matrix structure.

Sequencing studies have shown that *C. glabrata* is more closely related to *S. cerevisiae* than to *C. albicans* [89], with some genes functionally interchangeable among the two species [90,91]. An important component of the cell wall and the biofilm matrix is β-1,6-glucan, which is regulated by several genes, such as *KNH1.* Preceding studies have demonstrated that the *KNH1* homologs are essential components of β-1,6-glucan synthesis in *C. glabrata* [35,36,38]. In *S. cerevisiae*, many genes involved in β-1,6-glucan synthesis were isolated through mutations (*kre* [killer resistant] mutations) that are responsible for the resistance to the K1 killer toxin, which kills sensitive yeast cells after binding to β-1,6-glucan [35,38,92]. Dijkgraaf and colleagues [35] reported that the disruption of both *KRE9* and *KNH1* was synthetically lethal for *C. glabrata*, demonstrating the importance of these genes in the maintenance of cell structure. In the present study, after a drug stress, all *C. glabrata* strains upregulated this gene (Figure 1 and Table 3), indicating an effort to replace these β-1,6-glucans after losses due to the aggression of the antifungals, confirming also a certain degree of relevance of these elements in the cell wall and biofilm matrix of *C. glabrata* [35,36,38]. *C. glabrata* ATCC2001 showed to upregulate the *KNH1* gene in the presence of antifungal drugs, and *C. glabrata* 562123 indicated an identical pattern by marginally increasing *KNH1* gene expression in these conditions. Compared to the other two strains, with the exception of Flu treatment (FC: 2.08; *p* < 0.0001), *KNH1* showed a different regulation in the vaginal tract strain (Figure 1 and Table 3). *C. glabrata* 534784 demonstrated to have the highest upregulation capacity, presenting overexpression almost for all genes (Figure 1 and Table 3). 

During glucose starvation, a set of genes orthologous to *S. cerevisiae* is induced in *C. glabrata*, including *UGP1*, related to the β-1,6-d-glucan biosynthetic process [39,40], which shows that the environmental stress response is conserved between *S. cerevisiae* and *C. glabrata* [39]. *UPG1* showed to have the lowest expression, compared with other genes and controls. Nonetheless, except for one condition and strain, in the presence of antifungal drugs, several overexpression states were observed (Figure 1 and Table 3). The reference strain displayed overexpression in all conditions, with the highest gene upregulation occurring in the presence of AmB (FC: 0.17; *p* < 0.0001) and the lowest in the presence of Csf (FC: 0.01; non-significant); the urinary tract strain also revealed limited gene upregulation in the presence of Csf and AmB and the highest expression in the presence of Mcf (0.15; *p* < 0.0001). Generally, *C. glabrata* 534784 demonstrated the highest FC expression in all conditions. The lowest gene upregulation was observed in biofilm cells stressed by Csf (FC: 0.33; *p* < 0.0001). Srikantha and colleagues [91] identified a set of genes that are upregulated by the transcription factor Bcr1, involved in impermeability, impenetrability, and drug resistance of *C. albicans*’ biofilms. The authors concluded that the induction of Bcr1 overexpression in weak biofilms of *C. albicans* conferred those three characteristics and, in these cases, *UGP1* gene was downregulated [91]. This result supports the FC expression we obtained: since *C. glabrata* biofilms were weakened by the drugs, *UGP1* expression was increased in order to balance this defect (as seen with *KNH1*). The overexpression values we obtained for both *KNH1* and *UPG1* point to the relevance of β-1,6-glucans in the maintenance of a good cell and matrix structure.

Regarding mannans regulation, all strains showed a low or moderate expression of *MNN2* in the controls (non-treated cells), but relevant expression changes arose in the presence of all drugs, particularly when Mcf was added (Figure 1 and Table 3). *C. glabrata* ATCC2001 demonstrated the lowest expression in the control group, among all strains (Figure 1 and Table 3). The urinary strain presented the lowest gene expression, when compared to the untreated group (Figure 1 and Table 3). Flu and Mcf induced the highest *MNN2* values (FC: 0.34 and 1.21, respectively, both *p* < 0.0001), while AmB and Csf were associated with the lowest expressions (FC: 0.24, *p* < 0.0001 and 0.13, *p* < 0.0005). For *C. glabrata* ATCC2001 and *C. glabrata* 534784, the weakest effects were associated with the biofilm cells that were stressed by AmB (FC: 0.13 and 0.71 respectively, both *p* < 0.0001). When Mcf was applied, the biofilm cells of the vaginal strain showed a strong response to the stress, compared to the other two strains (FC: 7.03; *p* < 0.0001). Our team has also found that *C. glabrata* ATCC2001 increased the amounts of mannans on its cell walls in the presence of these drugs (data not shown), revealing a possible adaptation of the cells to the stress caused by the antifungal drugs. Other studies reported analogous adjustments of the cell walls after environmental drug stress, which has been related to high antifungal resistance events [1,2,19,92,93,94], supporting these results. 

Interestingly, and when compared to the rest of the genes, the present results demonstrate that *KNH1*, *UGP1*, and *MNN2* had the lowest values of FC expression. This seems to indicate that, although β-1,6-glucans are an important part of the cell and biofilm matrix, the cells appear to invest more in replacing the lost β-1,3-glucans, leading to consider that these components have a greater significance in the maintenance of the homeostasis of the biofilm matrix and the biofilm cells. In fact, in studies developed in our group [13,18], the total polysaccharides and β-1,3-glucans concentrations increased significantly in *C. glabrata* biofilm matrices after Flu, AmB, and Mcf contact. These higher concentrations in β-1,3 glucans content might explain part of the main biofilm resistance to the drugs that was formerly described [7,95,96,97]. 

Finally, downregulation of most genes and strains happened in the presence of AmB whereas, in opposition, Mcf induced the main overexpression alterations (Figure 1 and Table 3). AmB is a fungicidal drug and the most important antifungal polyene used for the treatment of systemic fungal infections [98,99]. This drug binds to the ergosterol of the cell membrane but also induces oxidative stress. This explains the existence of a still low reported rate of resistance and the good effectiveness of AmB [1,13,100,101,102,103,104]. Also, this low resistance may be associated with the lower gene expression effects that we detected after AmB exposure in *C. glabrata* (Figure 1 and Table 3). In opposition, the most acute upregulations occurred in the presence of echinocandins and, particularly, when Mcf was applied. This class of antifungals act by inhibiting β-1,3-glucan synthesis [1,100,105], which affects cell wall and matrix composition. By overexpressing the genes related to β-1,3-glucan synthesis (*BGL2*, *FKS1*, *FKS2*, *GAS2*, *XOG1*), the cells were attempting to compensate and replace the β-1,3-glucan losses in their matrices induced by the drugs and, thus, protect and decrease their susceptibility to the antifungals [19]. This general increase in total carbohydrates and specifically in β-1,3-glucans in *Candida* spp. biofilm matrices has already been described [7,13,18].

Regarding the correlation between the gene expression profiles in *C. glabrata*, the results based on the r are displayed in Table 4.

The results showed a strong positive correlation (r near 1) between the response profiles of *BGL2*, *XOG1*, *FKS1*, and *MNN2* gene expression in the three strains, which means that up and downregulation had a high tendency to occur similarly in all strains. The scores of the r for the profile of the *FKS2* gene revealed a moderate positive correlation between the reference strain (*C. glabrata* ATCC2001) and the isolates (*C. glabrata* 562123 and *C. glabrata* 534784). This indicates that, although the correlation was positive, it was weak, and the profiles of the gene response were variable in the three strains. On the other hand, the clinical isolates showed strong positive correlation between the expression profiles of this gene. *C. glabrata* ATCC2001 demonstrated a moderate positive correlation between the expression profiles of the *GAS2* gene. *C. glabrata* 562123 and *C. glabrata* 534784 had a strong positive correlation between the expression profiles of the *GAS2* gene. *KNH1* gene was the most variable and difficult gene to correlate between the strains. The reference and the 562123 strain showed a moderate correlation, whereas the reference and the 534784 strain showed a strong correlation, and the clinical isolates showed the only weak correlation detected in this study. As for the *UPG1* gene, although there was a negative correlation, the association between its expression in ATCC2001 and in the clinical isolates can be considered weak. Between the isolates, it was determined that *UPG1* up and downregulation had a high tendency to occur similarly in all strains (thus showing strong correlation). In summary, *BGL2*, *XOG1*, *FKS1*, and *MNN2* appeared to be the genes presenting the most similar responses to antifungal drugs within the transcriptome of the three strains; also, the clinical isolates appeared to be nearer each other than to the reference strain. Once more, β-1,3-glucan synthesis was identified as important in *C. glabrata* (three of the four genes affected are responsible for β-1,3-glucan production). These similarities among the two clinical strains may be due to the fact that both were derived from a hospital environment, and it is probable that they had already been challenged by several drugs, so their responses were prompter compared to the reference strain that is a wild type strain.

## 4. Conclusions

The in vitro high-dose paradox associated with *Candida* spp. isolates is being increasingly reported and connected to slightly elevated MICs, potentially contributing to clinical resistance and failure of antifungal treatments. These drug tolerance and adaptive mechanisms are highly related to *Candida* spp. biofilm forms. *C. glabrata* extracellular matrix is crucial for mature biofilm formation, not only contributing to the adhesive nature of the biofilm cells, but also protecting the cells from antifungal agents and from the host immune system. Understanding the production of the biofilm matrix components and the associated delivery processes is important for the development of effective biofilm therapies. All stakeholders in this process represent potentially attractive targets for detection of and therapeutic interventions against candidiasis.

## Figures and Tables

**Table 1:** Minimum biofilm eradicatory concentrations (MBEC) for the *Candida glabrata* strains of fluconazole (Flu), amphotericin B (AmB), caspofungin (Csf), and micafungin (Mcf) (mg/L).

Origin	Strain	Flu	AmB	Csf	Mcf
Reference (Wild Type)	ATCC2001	**>1250**	**4**	2.5–**3**	16–**17**
Urinary Tract	562123	**625**	**2**	0.5–**1**	16–**17**
Vaginal Tract	534784	**>1250**	**2**	2.5–**3**	5.5–**6**

**Bold**: concentrations applied to the pre-formed biofilms.

**Table 2:** Primers, targets used, and specific function of the genes used for the expression analysis.

Sequence (5′ → 3′)	Primer	Target	Properties and Proposed Function ^a^
5′-GGC AAG AAA CTG GAC AGA GC-3′	F	*BGL2*	β-1,3-glucanosyltransferase activity; glucan endo-β-1,3-d-glucosidase activity
5′-GGA AAA CTT GGG TCC TGC TG-3′	R		
5′-GTC CTA ACC TTG CAC ACC AG-3′	F	*FKS1*	β-1,3-d-glucan synthase activity
5′-CTA CGC CCA AAC ATC AGC-3′	R		
5′-GGG TCA CTG TGA AAT GTT-3	F	*FKS2*	β-1,3-d-glucan synthase activity
5′-GTA GAC GGG TTC GGA TT-3	R		
5′-ACC AGT CGT ACC ATT ACC GG-3′	F	*GAS2*	β-1,3-glucanosyltransferase activity
5′-CCT GCC CAA CTT CTA ACA GC-3′	R		
5′-CGG TGC CAA CGG TTA CTA-3′	F	*KNH1*	β-1,6-d-glucan biosynthetic process
5′-GTG ACA CGG GTT TCA GGA-3′	R		
5′-AAT CGC ACA AGG CAG AGA-3′	F	*UGP1*	β-1,6-d-glucan biosynthetic process
5′-ACT TGG GCG ACT TCC AAT-3′	R		
5′-GGT GAG TTG CAA CGT GAC AT-3′	F	*XOG1*	Glucan endo-β-1,6 and 1,3-glucosidase activity
5′-ATT CGG TTA AAG CGG CAC TC-3′	R		
5′-GAA GCC TGA TGG TGG TGA-3′	F	*MNN2*	α-mannosyltransferase biosynthetic process
5′-ATT GGG CGA TGA CCT TCT-3′	R		
5′-GTT GAC CGA GGC TCC AAT GA-3′	F	*ACT1*	Housekeeping gene
5′-CAC CGT CAC CAG AGT CCA AA-3′	R		

^a^ CGD: Candida Genome Database [63]; F: forward; R: reverse.

**Table 3:** Real-time PCR expression profiling of *BGL2*, *FKS1*, *FKS2*, *GAS2*, *KNH1*, *UGP1*, *XOG1*, and *MNN2* genes in biofilm cells of *C. glabrata* ATCC2001, *C. glabrata* 562123, *C. glabrata* 534784 with and without antifungal treatment (FC: 2^−ΔC_T_^). The significance of the FC results was determined by comparing the treated groups with the non-treated ones (controls) (* *p* < 0.05; ** *p* < 0.001; *** *p* < 0.0005; **** *p* < 0.0001).

Gene		*Candida glabrata* ATCC2001	*Candida glabrata* 562123	*Candida glabrata* 534784
		Fold-Change		
***BGL2***	Non treated	2.23	2.77	1.90
	Flu	1.41 ****	4.01 ****	3.66 ****
	AmB	2.04 ****	1.71 ****	3.85 ****
	Csf	2.00 ****	3.82 ****	5.98 ****
	Mcf	5.13 ****	10.58 ****	13.49 ****
***XOG1***	Non treated	0.57	0.15	0.46
	Flu	0.96 ****	0.54 ****	2.35 ****
	AmB	0.37 ****	0.10 ***	0.27 ****
	Csf	1.08 ****	1.42 ****	5.45 ****
	Mcf	6.54 ****	7.89 ****	12.38 ****
***GAS2***	Non treated	5.34	1.67	1.39
	Flu	0.76 ****	2.73 ****	2.26 ****
	AmB	3.02 ****	1.56 ****	1.95 ****
	Csf	5.72 ****	2.08 ****	3.39 ****
	Mcf	3.99 ****	7.43 ****	8.18 ****
***FKS1***	Non treated	0.11	0.11	0.17
	Flu	0.22 ****	0.65 ****	1.07 ****
	AmB	0.16 ****	0.08 ****	0.19 (ns)
	Csf	0.07 ***	0.20 ****	1.27 ****
	Mcf	0.49 ****	0.94 ****	3.55 ****
***FKS2***	Non treated	0.14	0.20	0.27
	Flu	0.61 ****	0.64 ****	1.77 ****
	AmB	0.06 ***	0.19 *	1.05 ****
	Csf	0.28 ****	0.55 ****	1.66 ****
	Mcf	0.43 ****	2.29 ****	7.50 ****
***KNH1***	Non treated	0.06	0.20	0.22
	Flu	0.50 ****	0.41 ****	2.08 ****
	AmB	0.51 ****	0.87 ****	0.94 ***
	Csf	0.24 ****	0.21 (ns)	0.72 ****
	Mcf	0.45 ****	1.33 ****	1.43 ****
***UGP1***	Non treated	0.002	0.07	0.20
	Flu	0.10 ****	0.15 ****	0.55 ****
	AmB	0.17 ****	0.05 ****	0.46 ****
	Csf	0.01 (ns)	0.06 **	0.33 ****
	Mcf	0.04 **	0.21 ****	3.17 ****
***MNN2***	Non treated	0.02	0.18	0.13
	Flu	0.19 ****	0.34 ****	1.17 ****
	AmB	0.13 ****	0.24 ****	0.71 ****
	Csf	0.18 ****	0.13 ***	1.40 ****
	Mcf	0.31 ****	1.21 ****	7.03 ****

(ns, non-significant; Non-treated, controls).

**Table 4:** Pearson Correlation Coefficient (r) determined for the expression profiles of *BGL2*, *FKS1*, *FKS2*, *GAS2*, *KNH1*, *UGP1*, *XOG1*, and *MNN2* genes in biofilm cells of *C. glabrata* ATCC2001, *C. glabrata* 562123, *C. glabrata* 534784, in the presence or absence of antifungal drugs.

Gene	ATCC2001 vs. 562123	ATCC2001 vs. 534784	562123 vs. 534784
*BGL2*	0.9100	0.9145	0.946
*XOG1*	0.9965	0.9459	0.9646
*FKS1*	0.8947	0.8723	0.8778
*FKS2*	0.5074	0.4481	0.9937
*GAS2*	−0.0514	0.1091	0.9663
*KNH1*	0.6427	0.8107	0.3697
*UGP1*	−0.1091	-0.1122	0.8519
*MNN2*	0.7924	0.8618	0.9728
